# LINC01224 promotes colorectal cancer progression through targeting miR-485-5p/MYO6 axis

**DOI:** 10.1186/s12957-021-02389-x

**Published:** 2021-09-17

**Authors:** Jingfeng Gu, Liang Dong, Yun Wang, Wenjia Nie, Wencong Liu, Ji-an Zhao

**Affiliations:** 1 Department of General Surgery, the First Hospital of Hebei Medical University, No.89 Donggang Road, Shijiazhuang, Hebei China; 2 Department of Medical Service, the First Hospital of Hebei Medical University, Shijiazhuang, China; 3 Department of Emergency, the First Hospital of Hebei Medical University, Shijiazhuang, China; 4 Department of Ultrasonography, the First Hospital of Hebei Medical University, Shijiazhuang, China

**Keywords:** Colorectal cancer, YY1, LINC01224, miR-485-5p, MYO6

## Abstract

**Background:**

Long noncoding RNAs (lncRNAs) are related to colorectal cancer (CRC) development. However, the role and mechanism of lncRNA LINC01224 in CRC development are largely unknown.

**Methods:**

LINC01224, Yin Yang 1 (YY1), microRNA (miR)-485-5p, and myosins of class VI (MYO6) levels were examined using quantitative reverse transcription polymerase chain reaction and western blotting. Functional analyses were processed through CCK-8, colony formation, flow cytometry, transwell, and xenograft analyses. Dual-luciferase reporter, chromatin immunoprecipitation (ChIP), RNA immunoprecipitation, and pull-down assays were conducted to analyze the binding interaction.

**Results:**

LINC01224 abundance was elevated in CRC tissue samples and cell lines. Elevated LINC01224 might indicate the lower 5-year overall survival in 52 CRC patients. LINC01224 was upregulated via the transcription factor YY1. LINC01224 knockdown restrained CRC cell proliferation, migration, and invasion and increased apoptosis. MiR-485-5p was sponged by LINC01224, and miR-485-5p downregulation relieved the influence of LINC01224 interference on CRC progression. MYO6 was targeted via miR-485-5p and regulated via LINC01224/miR-485-5p axis. MiR-485-5p overexpression suppressed CRC cell proliferation, migration, and invasion and facilitated apoptosis. MYO6 upregulation mitigated the role of miR-485-5p. LINC01224 knockdown decreased xenograft tumor growth.

**Conclusion:**

YY1-induced LINC01224 regulates CRC development via modulating miR-485-5p/MYO6 axis.

**Supplementary Information:**

The online version contains supplementary material available at 10.1186/s12957-021-02389-x.

## Introduction

Colorectal cancer (CRC) is a commonly diagnosed cancer with a high fatality rate around the world [1]. More than 90% CRCs are adenocarcinoma developing from the glandular epithelial cells of the colon and rectum [2]. Although huge improvements have been made on the pathogenesis and treatment of CRC, the prevention and prognosis of CRC remain unsatisfactory. Hence, exploring novel targets for CRC therapy is necessary.

Long noncoding RNAs (lncRNAs; >200 nucleotides) with diverse cellular functions and lacking protein-coding potential are related to human cancer progression [3]. lncRNAs have become potential targets for the treatment of CRC [4]. Moreover, the roles played via lncRNAs in CRC might be associated with the interaction of microRNAs (miRNAs) and mRNAs [5]. The lncRNA LINC01224 exhibits an oncogenic role in multiple human malignancies [6–9], including CRC [10]. Nevertheless, the repertoire of LINC01224 in CRC progression is largely unclear.

Moreover, LINC01224 promotes carcinogenesis and tumor development through serving as endogenous sponge for microRNAs (miRNA), including miRNA (miR)-2467 [10]. miRNAs (<25 nucleotides) are classical and versatile noncoding RNAs, and their association with the development, prognosis, and therapy of CRC has been better-demonstrated [11, 12]. MiR-485-5p is suggested as a target for LINC01224 in epithelial ovarian cancer [8], and miR-485-5p could function as an important biomarker for CRC [13]; furthermore, the tumor-suppressive role of miR-485-5p has also been discovered in CRC cells via affecting functional genes [14, 15]. Nevertheless, whether miR-485-5p is relevant to LINC01224 role in CRC remains to be further validated.

Myosins of class VI (MYO6) is a unique actin-based motor protein, which has important roles in regulating multiple cellular processes [16]. Previous reports have suggested an oncogenic role of MYO6 in several human cancers [17–19]. More importantly, MYO6 might contribute to the development of CRC malignancy as well [20, 21]. However, whether MYO6 can interact with LINC01224 and miR-485-5p is uncertain.

Thereby, we hypothesized that LINC01224 could affect CRC progression through regulating miR-485-5p and MYO6. To confirm this, expression of LINC01224, miR-485-5p, and MYO6 was detected in CRC patients and cells, and cellular functions of LINC01224 and miR-485-5p were measured in CRC cells. Ultimately, the underlying regulatory mechanism was further confirmed via LINC01224/miR-485-5p/MYO6 competing endogenous (ceRNA) pathway and upstream regulation of the transcription factor Yin Yang 1 (YY1), a well-known transcription factor for lncRNAs [22–24]. This research might indicate a novel insight into the pathogenesis of CRC.

## Materials and methods

### Ethics statement

Each patient signed the written informed consent. This research was approved by the ethics committee of the first hospital of Hebei Medical University, and all procedures were processed following the guidelines of the Helsinki Declaration. Animal experiments were authorized via the Institutional Animal Care and Use Committee of the first hospital of Hebei Medical University and conducted under the National Institutes of Health.

### Bioinformatics analysis

Expression of LINC01224 and MYO6 in colon adenocarcinoma (COAD) and rectum adenocarcinoma (READ) was provided via The Cancer Genome Atlas (TCGA) database (http://www.cancer.gov/tcga) [25]. The binding sites of YY1 and LINC01224 promoter were predicted via JASPAR algorithms (http://jaspar.genereg.net/) [26]. The binding sequences of miR-485-5p in LINC01224 or MYO6 were searched using starBase 2.0 of The Encyclopedia of RNA Interactomes. platform (http://starbase.sysu.edu.cn/index.php) [27]. The correlation among LINC01224, miR-485-5p, and MYO6 was analyzed on starBase project (http://starbase.sysu.edu.cn/panGeneCoExp.php). The association between LINC01224 level and overall survival of CRC patients was analyzed on the Gene Expression Profiling Interactive Analysis (GEPIA) server (http://gepia.cancer-pku.cn/) [28].

### Patient tissue collection

The CRC and normal control (NC) tissues were harvested from 52 CRC patients in the first hospital of Hebei Medical University from 2011 to 2014. All participants did not receive other treatment before surgery. According to the median of LINC01224 level, these 52 CRC patients were divided into two groups: LINC01224^high^ (*n* = 26) and LINC01224^low^ (*n* = 26). The 5-year overall survival of 52 CRC patients was recorded and analyzed by the Kaplan-Meier survival curve. The correlation between LINC01224 expression and the clinical-pathological features of CRC patients is shown in Table 1.

**Table 1:** Correlation between LINC01224 expression and the clinical-pathological features of CRC patients

Characteristic	All cases (*n* = 52)	LINC01224 expression		*P*-value
		High (*n* = 26)	Low (*n* = 26)	
Age (years)				0.578
<60	24	11	13	
≥60	28	15	13	
Sex				0.773
Male	19	9	10	
Female	33	17	16	
Tumor size, cm				0.780
>5	29	14	15	
≤5	23	12	11	
Distant metastasis				0.012*
Absent	25	8	17	
Present	27	18	9	
Lymph node metastasis				0.005*
No	22	6	16	
Yes	30	20	10	
TNM stage				0.575
I–II	22	10	12	
III	30	16	14	

Chi-square test was used. *P <0.05

### Cell culture

CRC cell lines (SW480, HCT116, SW620, and LoVo) and normal colonic mucosa FHC cells were obtained from BeNa Culture Collection (Beijing, China) and maintained in Roswell Park Memorial Institute (RPMI)-1640 medium (Thermo Fisher Scientific, Waltham, MA, USA) plus 10% fetal bovine serum (Gibco, Grand Island, NY, USA) at 37°C and 5% CO_2_.

### Quantitative polymerase chain reaction (qPCR)

After lysing in Trizol (Applygen, Beijing, China), the lysates were applied for total RNA extraction as previously reported [29]. A total of 1 μg RNA was used for cDNA generation with a reverse transcription kit (Thermo Fisher Scientific). The complex of cDNA, SYBR (Vazyme, Nanjing, China), and primer pairs were used for qPCR with the amplification protocols: 94°C for 5 min, 35 cycles of 94°C for 15 s, and 60°C for 60 s. The primer sequences were provided via Genscript (Nanjing, China) and listed in Table 2. Using U6 or GAPDH as a housekeeping gene, the relative RNA level was analyzed by 2^−ΔΔCt^ [30].

**Table 2:** Primers for qPCR and oligonucleotides for transfection

	Primers (5′-3′)
LINC01224	Forward, 5′-ACGTGCACAGACAGCTAAGA-3′; Reverse, 5′-GGAGTGACGATCCCTGGTGT-3′
YY1	Forward, 5′-GGGCCCTTTGTCCTGGATAC-3′; Reverse, 5′-GTGGATGAGACCTAGCCAGC-3′
MYO6	Forward, 5′-CCTCTTCACTGGCCCTCATC-3′; Reverse, 5′-CGGGCTTTCCATCCTCCATT-3′
miR-485-5p	Forward, 5′-TCGGCAGGAGAGGCTGGCCGT-3′; Reverse, 5′-AGTGCGTGTCGTGGAGTC-3′
U6	Forward, 5′-TTCGGCAGCACATATACT-3′; Reverse, 5′-CGCTTCACGAATTTGCGTGTCA-3′
GAPDH	Forward, 5′-GCCACTAGGCGCTCAC-3′; Reverse, 5′-AGCATCGCCCCACTTGATT-3′
si-YY1	Sense: 5′-UUUACUACUUUAUCAAAACAU-3′
si-NC	Sense: 5′-AAGACAUUGUGUGUCCGCCTT-3′
sh-LINC01224#1	Sense: 5′-GCACCUAGGUGUUGUCUUA-3′;
sh-LINC01224#2	Sense: 5′-GGACGUGCACAGACAGCUA-3
sh-NC	Sense : 5′-AACAGUCGCGUUUGCGACUG-3′
miR-485-5p mimic	5′-AGAGGCUGGCCGUGAUGCCUUC-3′
miR-NC mimic	5′-UUCUCCGAACGUGUCACGU-3′
Anti-miR-485-5p	5′-GAAUUCAUCACGGCCAGCCUCU-3′
Anti-NC	5′-UGAGCUGCAUAGAGUAGUGAUU-3′

### Cell transfection

YY1 and MYO6 overexpression vectors were constructed with pcDNA3.1 vector (Thermo Fisher Scientific). The siRNA targeting YY1 (si-YY1), si-NC, shRNA for LINC01224 (sh-LINC01224#1 and sh-LINC01224#2), sh-NC, miR-485-5p mimic, miR-NC mimic, miR-485-5p inhibitor (anti-miR-485-5p), and anti-NC were obtained from Genepharma (Suzhou, China), and their sequences are listed in Table 2. SW620 and LoVo cells were transfected by using Lipofectamine 3000 (Thermo Fisher Scientific).

### Western blotting

Protein was obtained through RIPA (Beyotime, Shanghai, China). With concentration determination using BCA kit (Beyotime), 20 μg proteins were separated via electrophoresis with sodium dodecyl sulfate, and then electrophoretically transferred onto nitrocellulose membranes (Bio-Rad, Hercules, CA, USA), which were blocked with 3% bovine serum albumin (Solarbio). Next, special proteins on the membranes were immunoblotted with corresponding primary antibodies for MYO6 (ab230478, 1:500, Abcam, Cambridge, UK), YY1 (ab109228, 1:1,000, Abcam), or GAPDH (ab181602, 1:3,000, Abcam), and then with universal secondary antibody (ab205718, 1:10,000, Abcam) for 3 h. Eventually, an immunoblotting signal was developed by exposing to ECL reagent (Beyotime), and the blots were detected via Quantity One v4.6 (Bio-Rad) with GAPDH as control.

### Cell Counting Kit (CCK)-8 and colony formation assay

1 × 10^4^ cells of differently treated SW620 and LoVo cells were seeded in 96-well plates with four paralleled wells in each group. These cells were further cultured for 72 h and then added with 10% CCK-8 reagent (Beyotime) for 3 h. The optical density was determined at 450 nm on a microplate reader (Bio-Rad). Cell viability was calculated by the relative OD value with normalization to the control group (100%). For colony formation assay, 1000 cells/well of SW620 and LoVo cells were seeded in 6-well plates for 14-day cultivation. Single-cell-formed colonies were visualized and counted after crystal violet staining (Solarbio, Beijing, China).

### Flow cytometry (FCM)

For cell apoptosis analysis, a group of 1 × 10^5^ cells of SW620 and LoVo cells were harvested for apoptosis and cell cycle analysis respectively using Annexin V-fluorescein isothiocyanate (FITC) apoptosis kit (Abcam) and propidium iodide (PI) FCM kit (Abcam). Briefly, apoptotic cells and cells distributed in cell cycle phases were examined with a flow cytometer (Beckman Coulter, Fullerton, CA, USA).

### Transwell assay

Transwell assay was applied to detect the migratory and invasive abilities. For migration assay, 2 × 10^5^ cells of SW620 and LoVo cells were re-suspended in serum-free RPMI-1640 medium for migration assay with Transwell chamber (BD, Franklin Lakes, NJ, USA), and 5 × 10^5^ cells were for invasion assay with Transwell chamber (BD) coated with Matrigel. In brief, these cells were seeded in the upper chamber, and a complete medium was loaded in the lower chamber as an attractant. Transwell systems were incubated in normal cell culture condition for 24 h, and cells transferred onto the lower membranes were visualized and counted after crystal violet staining (Solarbio).

Cells were stained using 0.5% crystal violet. The images of migratory or invasive cells were captured under a microscope (Nikon, Tokyo, Japan) at magnification ×100.

### Dual-luciferase reporter, chromatin immunoprecipitation (ChIP), RNA immunoprecipitation (RIP), and pull-down assays

LINC01224 promoter containing YY1 binding sites (GCCATC) or mutant type (CGGTAG) were cloned in pGL3-basic vector (Promega, Madison, WI, USA) to generate pGL3-WT1 (pGL3-LINC01224-WT1), pGL3-MUT1 (pGL3-LINC01224-MUT1), pGL3-WT2 (pGL3-LINC01224-WT2), or pGL3-MUT2 (pGL3-LINC01224-MUT2), which were then transfected into SW620 and LoVo cells together with YY1 overexpression vector or vector. The sequences of LINC01224 or MYO6-3′UTR containing miR-485-5p target sites or mutant sites were cloned in pGL3-basic vector to generate LINC01224-WT, LINC01224-MUT, MYO6-3′UTR-WT, or MYO6-3′UTR-MUT, which were transfected in SW620 and LoVo cells together with miR-485-5p mimic or miR-NC. Luciferase activity was detected with a dual-luciferase analysis kit (Promega).

For the ChIP assay, a Magnetic ChIP Kit (Thermo Fisher Scientific) was used. 1 × 10^4^ SW620 and LoVo cells were fixed with 1% formaldehyde (Solarbio) and then were sonicated for cutting DNA into 500-bp fragments. The DNA samples were precipitated with YY1 antibody (anti-YY1) for 6 h with anti-IgG as control, in the presence of magnetic beads. The precipitated chromatin DNA was isolated for qPCR.

Cell lysates containing 1 × 10^7^ cells of SW620 and LoVo cells were collected and incubated with Protein A/G Sepharose® beads provided in the Magna RIP kit (Millipore, Billerica, MA, USA). These beads were pre-conjugated with the antibody of Ago2 (anti-Ago2) or anti-IgG for 8 h. Similarly, cell lysates containing 1 × 10^7^ cells which were pre-transfected with biotinylated miR-485-5p mimic (Bio-miR-485-5p), Bio-NC, biotin-labeled LINC01224 probe, or oligo probe were collected and incubated with the magnetic beads supplied by Magnetic RNA-Protein Pull-Down Kit (Thermo Fisher Scientific). Relative RNA expression levels in precipitated RNA complex on the beads were measured by qPCR.

### Xenograft experiment

sh-LINC01224#1 or sh-NC-transfected SW620 cells (5 × 10^6^) were subcutaneously injected in 5-week-old male BALB/c athymic mice (Vital River Laboratory Animal Technology, Beijing, China) (*n* = 6/group). The length and width of each xenograft tumor were monitored every 7 days, and tumor volume was calculated by 0.5 × length × width^2^ [31]. After 35 days, tumor-bearing mice were euthanized using 5% isoflurane, and the tumors were detached and weighted. And, tumor tissues were lysed to detect relative levels of LINC01224, miR-485-5p, and MYO6.

### Statistical analysis

Data were shown as mean ± standard deviation from three repeats. The linear correlation between two variables was tested by Pearson’s correlation analysis. Association between LINC01224 level and overall survival of patients was evaluated via Kaplan-Meier plot and log-rank test. Difference was analyzed by Student’s *t*-test or analysis of variance followed by Tukey’s post hoc test. Data analysis was performed on GraphPad Prism 6 (GraphPad Inc., La Jolla, CA, USA), and the significance of difference was set as *P* < 0.05.

## Results

### LINC01224 level is increased and activated by YY1 in CRC

**Fig. 1:**
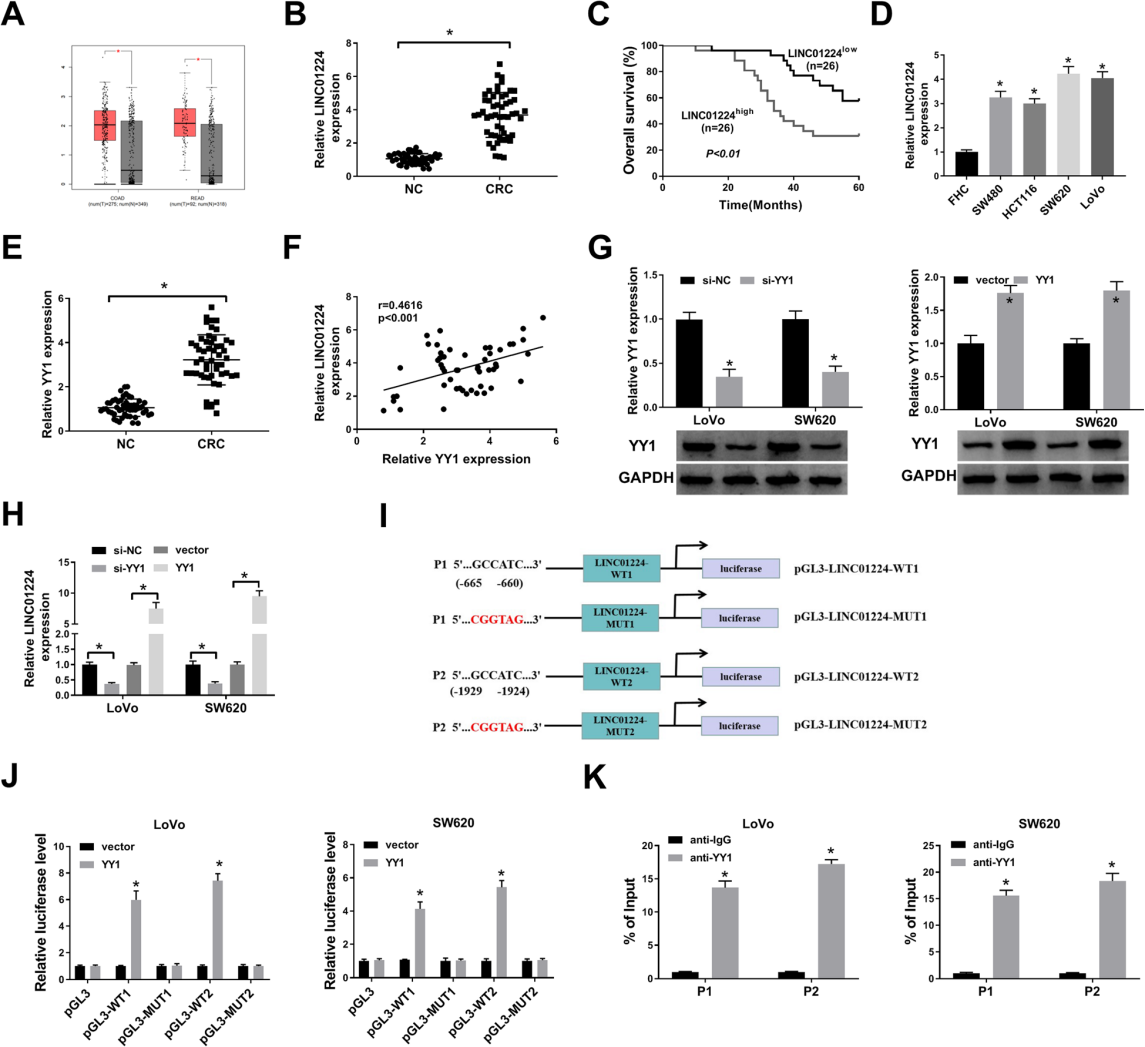
The expression of LINC01224 and the interaction between LINC01224 and YY1 in CRC. **A** LINC01224 level in colon adenocarcinoma (COAD) and rectum adenocarcinoma (READ) was predicted via GEPIA with normalization to TCGA normal and GTEx data. Log2FC>1; *P*-value > 0.01. **B** LINC01224 abundance was determined via qPCR in CRC and normal control (NC) samples (*n* = 52). **C** The overall survival of patients was evaluated in LINC01224^high^ and LINC01224^low^ groups. **D** LINC01224 expression was detected via qPCR in CRC and control cell lines. **E** YY1 abundance was measured via qPCR in CRC tissues and NC samples (*n* = 52). **F** The linear correlation between YY1 and LINC01224 levels in CRC tissues. **G** YY1 expression was tested via western blotting in LoVo and SW620 cells with transfection of si-NC, si-YY1, vector, or YY1 overexpression vector. **H** LINC01224 expression was determined via qPCR in cells transfected with si-NC, si-YY1, vector, or YY1 overexpression vector. **I** The construction of pGL3-LINC01224-WT1, pGL3-LINC01224-MUT1, pGL3-LINC01224-WT2, and pGL3-LINC01224-MUT2. **J** Luciferase activity of pGL3-WT1, pGL3-MUT1, pGL3-WT2, and pGL3-MUT2 was determined in LoVo and SW620 cells co-transfected with YY1 overexpression vector or its control vector. **K** The affinity of YY1 to LINC01224 promoter was analyzed via ChIP assay. ^*^P < 0.05

To study the importance of LINC01224 in CRC, the expression change of LINC01224 was detected. LINC01224 level was elevated in COAD and READ in the TCGA database (Fig. 1A). Furthermore, LINC01224 abundance was 4-fold higher in CRC than in NC tissues (*n* = 52) (Fig. 1B). Additionally, the patients were classified into LINC01224^high^ and LINC01224^low^ groups according to the median value of LINC01224. Clinically, patients with high LINC01224 expression had distant metastasis, lymph node metastasis, and poor overall survival (*P* < 0.01) (Table 1 and Fig. 1C). However, TCGA patients showed that LINC01224 expression might be not associated with overall survival in COAD and READ (Figure S1A). Moreover, LINC01224 abundance was evidently increased in CRC cell lines (SW480, HCT116, SW620, and LoVo) compared with the FHC cell line (Fig. 1D). In order to explore which resulted in the expression change of LINC01224, the upstream transcription factor was analyzed. YY1 was one of the well-known transcription factors for lncRNAs [22–24], and we predicted YY1 could bind to the promoter of LINC01224 using JASPAR algorithms. YY1 mRNA expression was higher in CRC tissues than in the NC group (Fig. 1E). Moreover, YY1 abundance in CRC was positively correlated with LINC01224 (Fig. 1F). In addition, we assessed whether YY1 could change LINC01224 abundance in LoVo and SW620 cells. The efficacy of si-YY1 and YY1 overexpression vector is confirmed in Fig. 1G. LINC01224 abundance was reduced 60% by YY1 knockdown and increased more than 7.5-fold by YY1 overexpression (Fig. 1H). Furthermore, we selected 2 different fragments containing the binding sites between YY1 and LINC01224 with the highest prediction scores (P1 and P2) (Fig. 1I). The dual-luciferase activity analysis showed that YY1 overexpression obviously enhanced the luciferase activity of pGL3-WT1 or pGL3-WT2 with the wild-type LINC01224 promoter region, but not the mutant type (Fig. 1J). Besides, the 2 fragments (P1 and P2) were responsible for the affinity of YY1 and LINC01224 (Fig. 1K). These data suggested that YY1 transcriptionally activated LINC01224 expression.

### LINC01224 silencing represses CRC progression in vitro

**Fig. 2:**
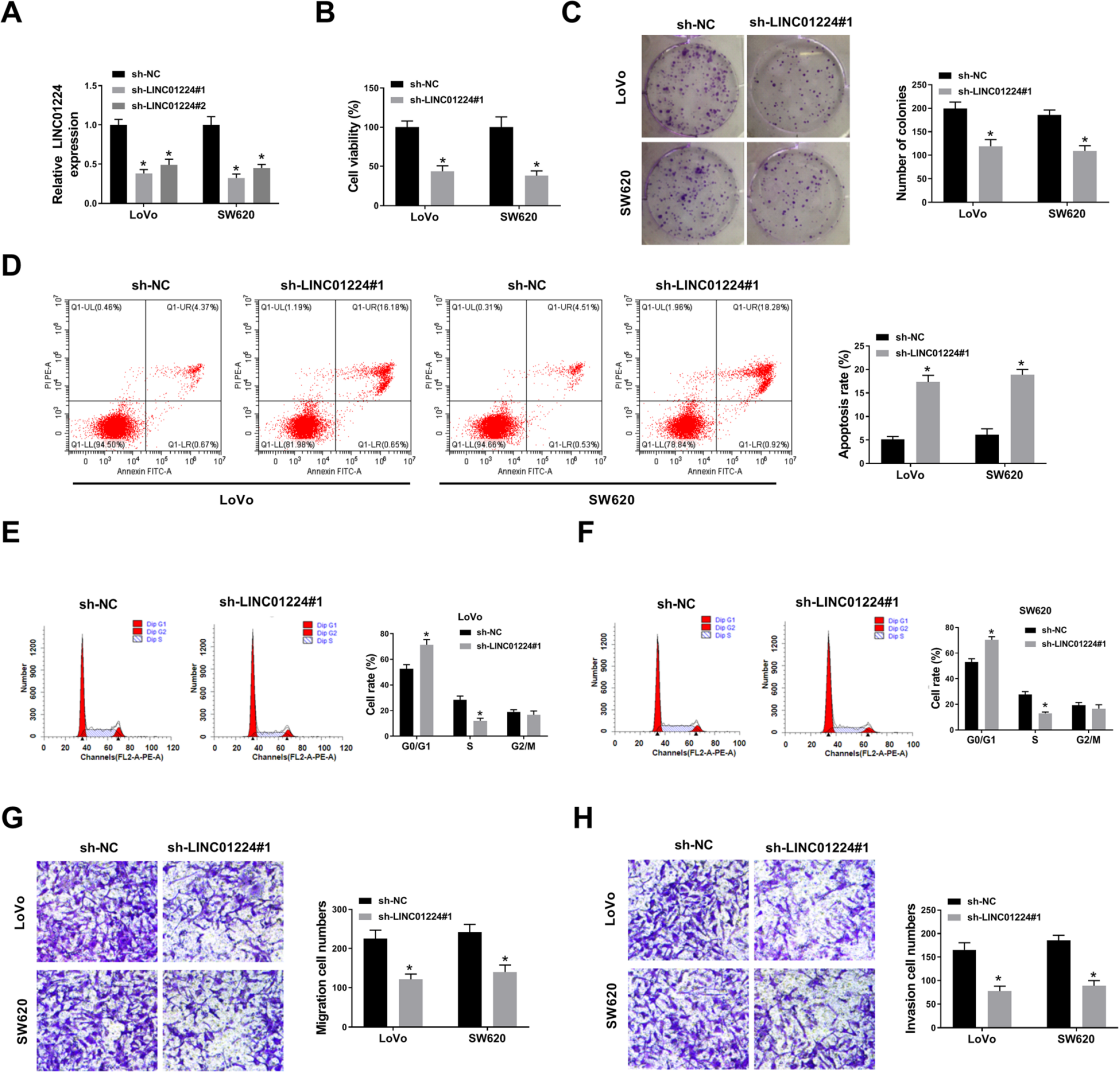
The role of LINC01224 in CRC progression. **A** LINC01224 abundance was measured in LoVo and SW620 cells with addition of sh-NC, sh-LINC01224#1, or sh-LINC01224#2. **B** Cell viability was examined via CCK-8 in cells with addition of sh-NC or sh-LINC01224#1 at 72 h. **C** Colony formation was tested in cells with addition of sh-NC or sh-LINC01224#1. **D** Cell apoptosis was tested through FCM in LoVo and SW620 cells with transfection of sh-NC or sh-LINC01224#1 at 72 h. **E**, **F** Cell cycle process was analyzed by FCM analysis in LoVo and SW620 cells with sh-NC or sh-LINC01224#1 transfection. **G**, **H** Migratory and invasive abilities were analyzed via transwell analysis in LoVo and SW620 cells transfected with sh-NC or sh-LINC01224#1. ^*^P < 0.05

To study the function of LINC01224 in CRC progression in vitro, LINC01224 abundance in LoVo and SW620 cells was exhausted via shRNAs. LINC01224 level was evidently declined via the transfection of sh-LINC01224#1 or sh-LINC01224#2 compared with sh-NC, and sh-LINC01224#1 with higher efficacy was used for further experiments (Fig. 2A). LINC01224 interference evidently reduced cell viability and colony formation ability of LoVo and SW620 cells (Fig. 2B, C). Moreover, LINC01224 silencing evidently induced cell apoptosis and arrested cell cycle (Fig. 2D–F). Additionally, LINC01224 interference clearly restrained the migration and invasion abilities of LoVo and SW620 cells (Fig. 2G, H). These data suggested LINC01224 knockdown constrained CRC progression in vitro.

### MiR-485-5p inhibition attenuates the impact of LINC01224 interference in CRC cells

**Fig. 3:**
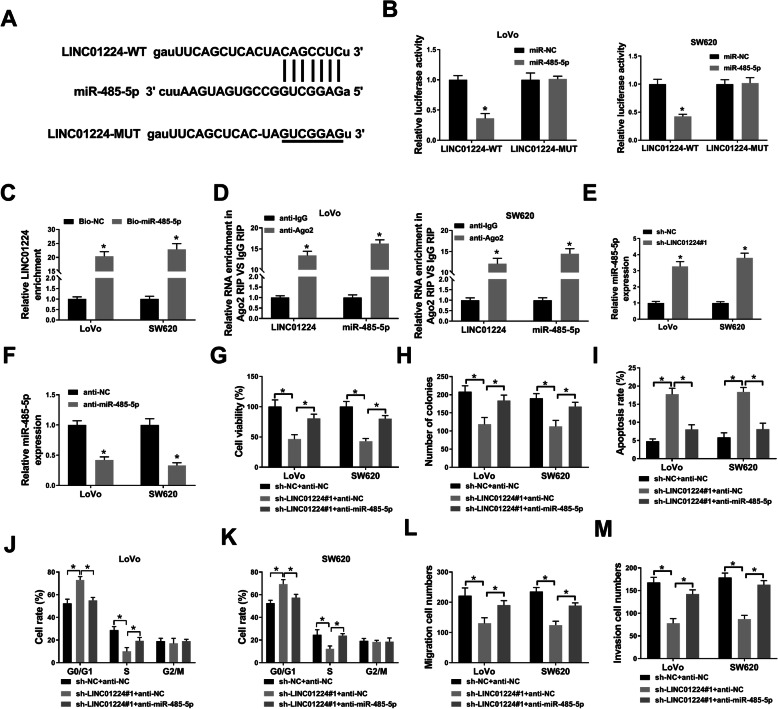
The relationship between LINC01224 and miR-485-5p in CRC cells. **A** The target sites of LINC01224 and miR-485-5p. **B** Luciferase activity of LINC01224-WT and LINC01224-MUT vectors was measured in LoVo and SW620 cells co-transfected with miR-485-5p mimic or miR-NC. **C** LINC01224 expression was measured after RNA pull-down using Bio-miR-485-5p or Bio-NC. **D** LINC01224 and miR-485-5p levels were detected after RIP of Ago2 or IgG. **E** MiR-485-5p level was determined in LoVo and SW620 cells transfected with sh-NC or sh-LINC01224#1. **F** MiR-485-5p abundance was determined in cells with transfection of anti-miR-485-5p or anti-NC. Cell viability (**G**), colony formation (**H**), apoptosis (**I**), cycle process (**J**, **K**), migration (**L**), and invasion (**M**) were measured by CCK-8 assay, colony formation assay, FCM, and transwell assay in LoVo and SW620 cells co-transfected with sh-NC + anti-NC and sh-LINC01224#1 + anti-NC or anti-miR-485-5p. ^*^P < 0.05

To probe the network modulated by LINC01224, the target miRNAs were searched via starBase, and a total of 11 potential target miRNAs for LINC01224 were obtained. Among these miRNAs, miR-485-5p was the most enriched by biotin-labeled LINC01224 probe in both LoVo and SW620 cells (Figure S2A and B). Thus, miR-485-5p was the most promising candidate as LINC01224 target in CRC. The predicted binding sequences between LINC01224 and miR-485-5p were mutated for luciferase assay (Fig. 3A), and miR-485-5p mimic-mediated overexpression could reduce the luciferase activity of LINC01224-WT but not LINC01224-MUT (Fig. 3B). Furthermore, RNA pull-down analysis displayed LINC01224 could bind with miR-485-5p (Fig. 3C). In addition, both LINC01224 and miR-485-5p were co-enriched by Ago2 RIP (Fig. 3D). Besides, the miR-485-5p level was obviously elevated via LINC01224 knockdown (Fig. 3E). These data indicated miR-485-5p was sponged by LINC01224. To study whether miR-485-5p was involved in LINC01224-mediated CRC development, rescue experiments were launched with co-transfection. The addition of anti-miR-485-5p lowered miR-485-5p abundance in LoVo and SW620 cells (Fig. 3F), and this downregulation reversed LINC01224 knockdown-mediated effects on cell viability, colony formation ability, apoptosis, and cell cycle (Fig. 3G–K). Furthermore, miR-485-5p inhibition improved migration and invasion abilities in LoVo and SW620 cells with LINC01224 interference (Fig. 3L, M). These findings indicated that LINC01224 modulated CRC progression via miR-485-5p.

### MiR-485-5p overexpression inhibits CRC progression via target inhibiting MYO6

**Fig. 4:**
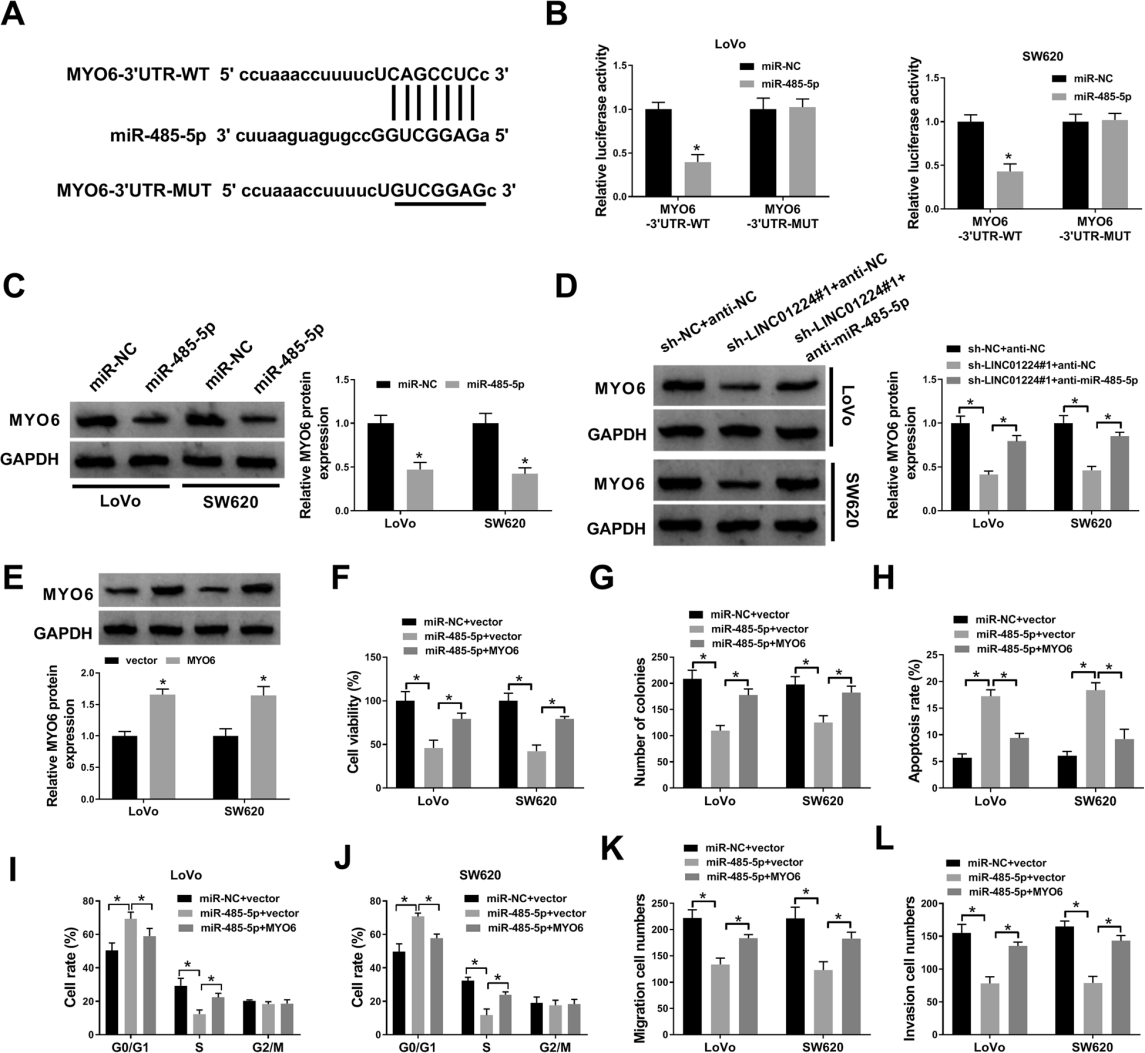
The target relationship between miR-485-5p and MYO6 in CRC cells. **A** The target sites of miR-485-5p and MYO6. **B** Luciferase activity of MYO6-3′UTR-WT and MYO6-3′UTR-MUT vectors was detected in LoVo and SW620 cells co-transfected with miR-485-5p mimic or miR-NC. **C**–**E** MYO6 protein level was determined by western blotting in LoVo and SW620 cells transfected with **C** miR-NC or miR-485-5p mimic, **D** sh-NC + anti-NC, sh-LINC01224#1 + anti-NC or anti-miR-485-5p, and **E** vector or MYO6 overexpression vector. Cell viability (**F**), number of colonies (**G**), apoptosis rate (**H**), cell rate in cell cycle phases (**I**, **J**), migration cell number (**K**), and invasion cell number (**L**) were measured by CCK-8 assay, colony formation assay, FCM and transwell assay in LoVo and SW620 cells co-transfected with miR-NC + vector and miR-485-5p mimic + vector or MYO6 overexpression vector. ^*^P < 0.05

To probe the mechanism mediated via LINC01224/miR-485-5p axis, the targets of miR-485-5p were searched using starBase. The target sequences (CAGCCUC) of miR-485-5p and MYO6 are exhibited in Fig. 4A. TCGA-COAD (colon adenocarcinoma) and READ (rectum adenocarcinoma) analysis show the overexpression of MYO6 in CRC tumors (http://www.cancer.gov/tcga) (Figure S1B). Moreover, our qPCR data showed the downregulation of miR-485-5p and the upregulation of MYO6 in 52 CRC tumors than paired normal tissues (Figure S1C and 1D). However, there seemed no correlation among LINC01224, miR-485-5p, and MYO6 levels in COAD patients integrated from the TCGA project (Figure S1E). To further validate the potential target relationship between miR-485-5p and MYO6, we performed a luciferase assay. As a result, miR-485-5p mimic addition led to a 60% decrease of luciferase activity of MYO6-3′UTR-WT, and no reduction on that of MYO6-3′UTR-MUT (Fig. 4B). Additionally, MYO6 protein expression was sharply decreased in response to miR-485-5p mimic transfection (Fig. 4C). Besides, the MYO6 protein level was markedly declined by LINC01224 interference, and it was enhanced by miR-485-5p inhibition (Fig. 4D). In order to study the function of miR-485-5p/MYO6 axis in CRC development in vitro, LoVo and SW620 cells were transfected with different contents. The MYO6 protein level was increased 1.6-fold by the addition of the MYO6 overexpression vector (Fig. 4E). Moreover, miR-485-5p upregulation evidently reduced cell viability and colony formation ability, and it was reversed by MYO6 elevation (Fig. 4F, G). Furthermore, miR-485-5p upregulation clearly promoted cell apoptosis and cycle arrest, and this effect was weakened by the introduction of MYO6 (Fig. 4H–J). Besides, miR-485-5p overexpression evidently declined cell migration and invasion, and this effect was abated via MYO6 addition (Fig. 4K, L). Collectively, miR-485-5p could repress CRC progression in vitro via regulating MYO6.

### LINC01224 knockdown reduces cell growth in vivo

**Fig. 5:**
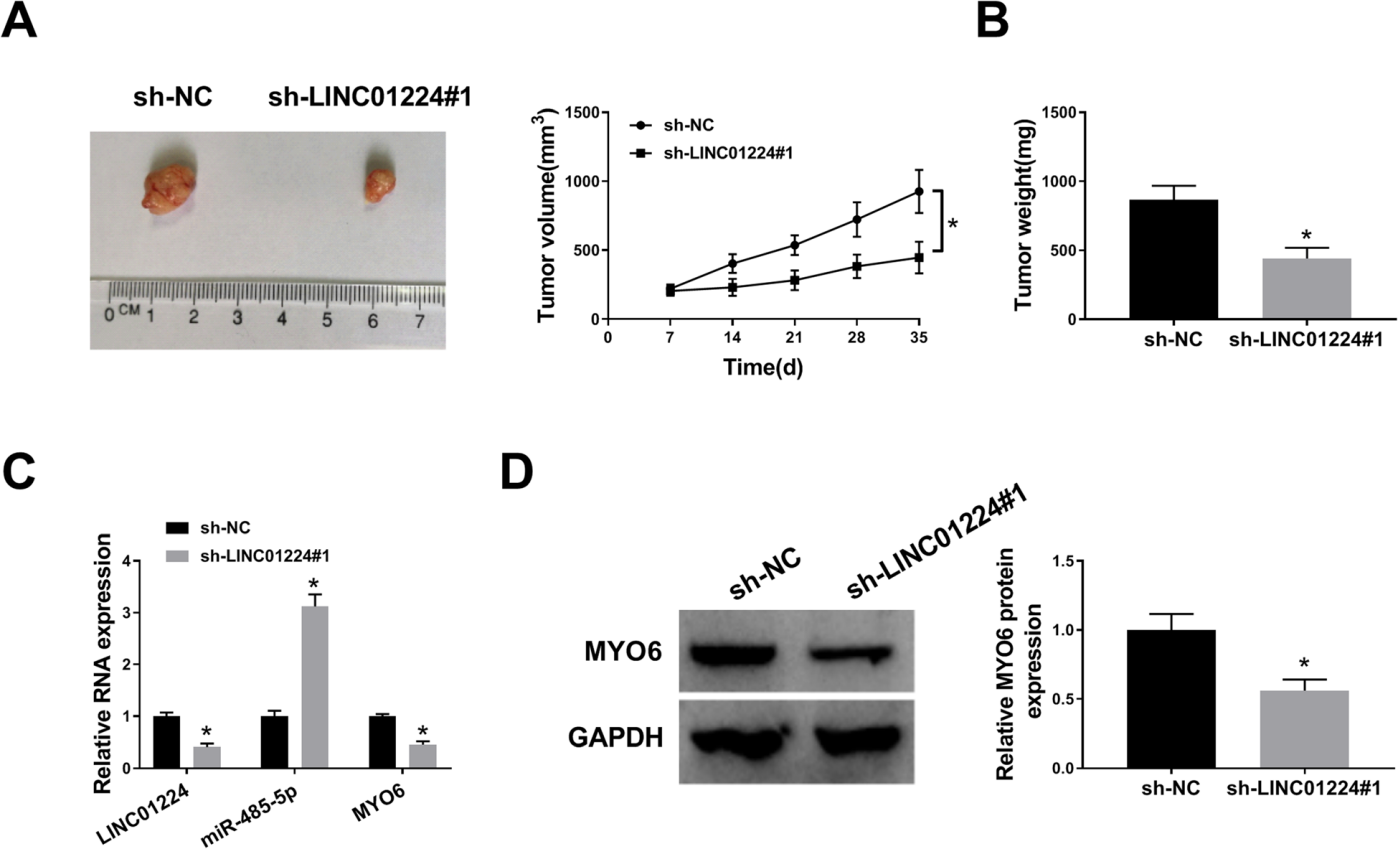
The influence of LINC01224 on CRC cell growth in vivo. **A**, **B** Tumor volume and weight were examined in each group. **C**, **D** LINC01224, miR-485-5p, and MYO6 levels were determined by qPCR and western blotting in tumor tissues. ^*^P < 0.05

To further study the role of LINC01224 in CRC progression, LINC01224-silenced SW620 cells were used to establish a xenograft tumor model in mice. After 35 days, the volume and weight of xenograft tumors were obviously lower in the sh-LINC01224#1 group comparing to the control group (Fig. 5A, B). Furthermore, LINC01224 and MYO6 abundances were markedly higher and miR-485-5p was lower in the tumor tissues with sh-LINC01224#1 transfection (Fig. 5C, D). These data showed that LINC01224 silencing reduced CRC cell growth in vivo.

## Discussion

CRC is a common malignancy with high incidence (~10% of all cancers) and mortality (~9% of all cancers) worldwide [32]. lncRNAs are related to cancer phenotypes in CRC via controlling cellular processes, including proliferation, apoptosis, migration, and invasion [33]. Mechanically, lncRNAs function as miRNA sponges and mediate competing endogenous (ceRNA) regulatory networks [34–36]. Here, we confirmed the role of LINC01224 in CRC progression and identified the ceRNA pathway of LINC001224/miR-485-5p/MYO6.

Additionally, there is a reported prognostic signature of 6-lncRNA and 12-gene for CRC [37, 38]. Like the data of the TCGA database, we also found LINC01224 abundance was elevated in CRC. Moreover, the increased LINC01224 indicated the poor outcomes of patients. To probe the role of LINC01224 in vitro, we measured the LINC01224 level in CRC cell lines and found that LINC01224 abundance was elevated in CRC cells. Next, we explored which led to the upregulation of LINC001224. YY1 is a zinc finger protein involved in transcriptional control in human cancers [39]. Moreover, YY1 is related to the regulation of lncRNAs [40]. The increasing evidence has reported that YY1 can activate the expression of lncRNA DDX11 antisense RNA 1 (DDX11-AS1), RAP1 antisense RNA 1 (ARAP1-AS1), and growth arrest-specific transcript 5 (GAS5) in CRC [41–43]. Here, we found that the transcription factor YY1 could bind and induce LINC01224 expression. Moreover, we found LINC01224 silencing constrained CRC cell proliferation and motility, but promoted apoptosis, indicating the anti-CRC role of LINC01224 silencing, which was consistent with the previous study [10].

lncRNA-mediated ceRNA regulatory network is an important mechanism in CRC [44]. Gong et al. have proposed a regulatory network of LINC001224/miR-330-5p/checkpoint kinase 1 (CHEK1) in hepatocellular carcinoma [7]. Chen et al. claimed that LINC001224 sponged miR-2467 to promote CRC progression [10]. To explore an additional regulatory network, we validated miR-485-5p could be sponged via LINC01224. Multiple reports suggested miR-485-5p suppresses cancer progression of CRC [13, 14, 45, 46]. Similarly, our study also found that miR-485-5p addition could repress CRC progression. Additionally, miR-485-5p inhibition abated the cellular functions of LINC01224 depletion on CRC progression, implying that LINC01224 regulated CRC progression via mediating miR-485-5p.

Moreover, we further identified MYO6 was targeted via miR-485-5p. Previous studies have indicated that MYO6 can facilitate CRC malignancy via boosting cell viability, cell cycle, cell motility, and glycolysis [20, 21, 47–50]. These reports suggested that MYO6 played an oncogenic role in CRC. Our research also validated the function of MYO6 via rescuing miR-485-5p-mediated suppressive role in CRC progression. Furthermore, we found that MYO6 expression was regulated via LINC01224 and miR-485-5p, suggesting that LINC01224 could target MYO6 with miR-485-5p as a crosstalk. The preclinical models are helpful to understand the pathogenesis of CRC [51]. Hence, to better expound LINC01224 role in CRC, we established the subcutaneously xenograft model using CRC cells. We found that LINC01224 knockdown decreased xenograft tumor growth, indicating the anti-CRC role of LINC01224 knockdown in vivo.

However, there are still some limitations in our study. Firstly, the sample size is 52 for the 5-year overall survival analysis of CRC patients. Secondly, starBase predicts other miRNAs and genes, which can be targeted by LINC01224 and miR-485-5p, and other pathways in CRC progression can be explored.

In conclusion, LINC01224 knockdown represses CRC progression both in vitro and in vivo partly by regulating miR-485-5p and MYO6 via the ceRNA axis. Our study deepens our understanding on CRC pathogenesis. The study also indicates that LINC01224 high level may act as an unfavorable marker for the overall survival of these 52 CRC patients.

## Supplementary Information

**Additional file 1: Figure S1.** The enrichment of miRNAs in biotin-labelled LINC01224 probe in CRC cells. (A, B) RNA pull-down assay and qPCR determined relative miRNA expression in LINC01224 probe and oligo probe-mediated pull-down contents in LoVo and SW620 cells. ^***^P < 0.001.

**Additional file 2: Figure S2.** Expression analysis of LINC01224, miR-485-5p and MYO6 in CRC patients. (A) GEPIA database showed the overall survival of COAD and READ patients (integrated from TCGA project) with High and Low LINC01224 level. (B) MYO6 level in COAD and READ was predicted via GEPIA with normalization to TCGA normal and GTEx data. Log2FC>1; *p*-value>0.01. (C, D) Relative miR-485-5p and MYO6 expression was determined via qPCR in CRC and normal control (NC) samples (*n* = 52). (E) StarBase 3.0 project analyzed the correlation among LINC01224, miR-485-5p and MYO6 levels in CRC patients (integrated from TCGA project). ^*^P < 0.05.

## Data Availability

Not applicable.

## Abbreviations

lncRNAs: Long noncoding RNAs
CRC: Colorectal cancer
YY1: Yin Yang 1
MYO6: Myosins of class VI
ChIP: Chromatin immunoprecipitation
