# Supplementary figures and images for: LINC01224 promotes colorectal cancer progression through targeting miR-485-5p/MYO6 axis
Source: World J Surg Oncol. 2021 Sep 17;19:281. doi: 10.1186/s12957-021-02389-x (PMC8449439; doi:10.1186/s12957-021-02389-x)

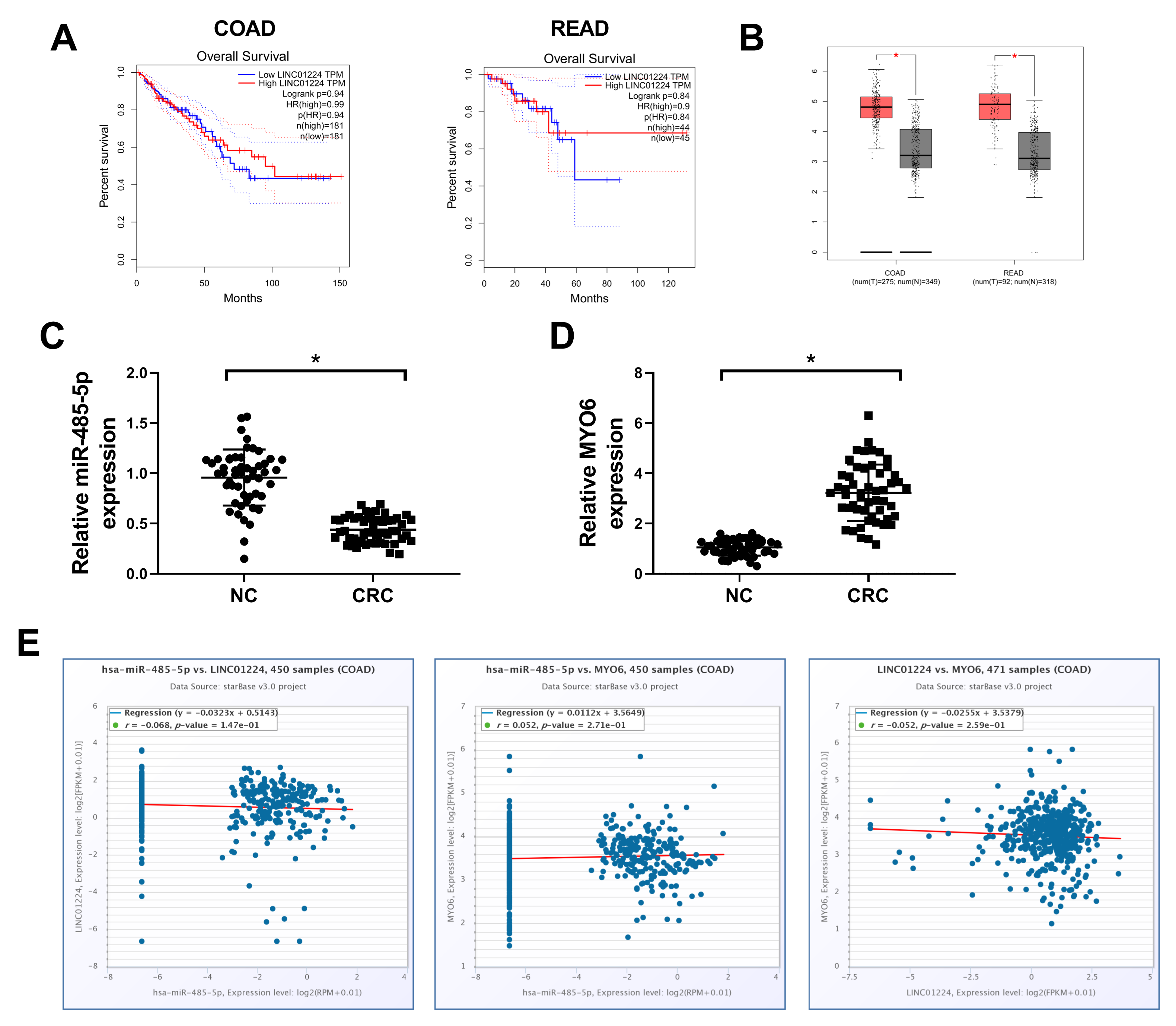

Supplement: Supplementary file 1 — Additional file 1: Figure S1. The enrichment of miRNAs in biotin-labelled LINC01224 probe in CRC cells. (A, B) RNA pull-down assay and qPCR determined relative miRNA expression in LINC01224 probe and oligo probe-mediated pull-down contents in LoVo and SW620 cells. ***P < 0.001. [file 12957_2021_2389_MOESM1_ESM.tif]

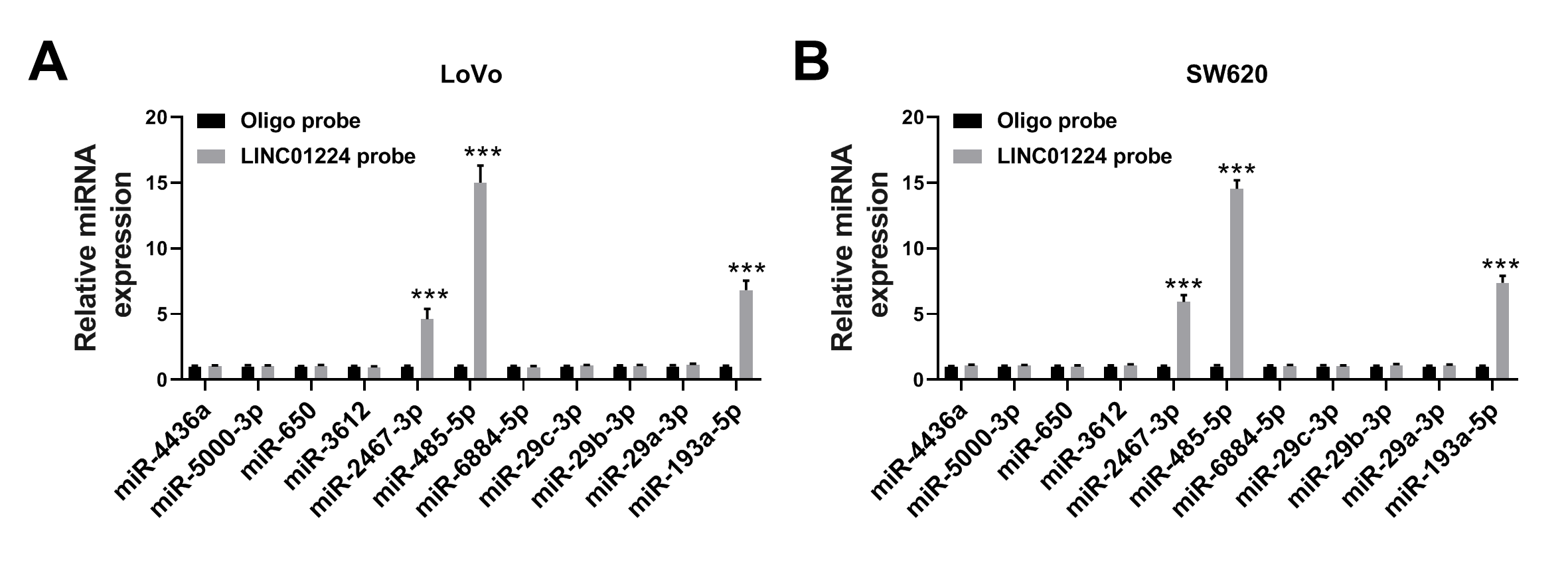

Supplement: Supplementary file 2 — Additional file 2: Figure S2. Expression analysis of LINC01224, miR-485-5p and MYO6 in CRC patients. (A) GEPIA database showed the overall survival of COAD and READ patients (integrated from TCGA project) with High and Low LINC01224 level. (B) MYO6 level in COAD and READ was predicted via GEPIA with normalization to TCGA normal and GTEx data. Log2FC>1; p-value>0.01. (C, D) Relative miR-485-5p and MYO6 expression was determined via qPCR in CRC and normal control (NC) samples (n = 52). (E) StarBase 3.0 project analyzed the correlation among LINC01224, miR-485-5p and MYO6 levels in CRC patients (integrated from TCGA project). *P < 0.05. [file 12957_2021_2389_MOESM2_ESM.tif]
